# The Slovenian Nutrition Guidelines 2025: A Comparison with the Prior Slovenian FBDG, Dietary Intake, and the EAT–Lancet Diet

**DOI:** 10.3390/foods15030524

**Published:** 2026-02-03

**Authors:** Nataša Fidler Mis, Boštjan Jakše, Zlatko Fras

**Affiliations:** 1 Independent Researcher, 1000 Ljubljana, Slovenia; 2 Independent Researcher, 4280 Kranjska Gora, Slovenia; bj7899@student.uni-lj.si; 3 Division of Medicine, Centre for Preventive Cardiology, University Medical Centre Ljubljana, 1000 Ljubljana, Slovenia; zlatko.fras@kclj.si; 4 Faculty of Medicine, University of Ljubljana, 1000 Ljubljana, Slovenia

**Keywords:** public health nutrition, Slovenian Nutrition Guidelines 2025, EAT–Lancet diet, food-based dietary guidelines, dietary intake surveillance, Slovenia, diet alignment, legumes, red/processed meat, sodium, trans fatty acids, ultra-processed foods, free sugars, sex specific

## Abstract

**Background**: The Slovenian Nutrition Guidelines 2025 (SNG2025) provide a quantified, plant-forward framework aligned with the EAT–Lancet diet, whereas previous Slovenian FBDGs were qualitative. **Objectives**: (i) To compare SNG2025 with the EAT–Lancet diet and previous Slovenian FBDGs and (ii) to assess the alignment of food intake among Slovenian adults with the SNG2025. **Methods**: The SNG2025 food group targets were mapped to the EAT–Lancet diet and previous Slovenian FBDGs and evaluated against a nationally representative intake (Si. Menu 2017/18; 18–64 years; sex-specific). Sodium intake was corroborated by 24-h urinary sodium levels (2022). **Results**: The SNG2025 introduces numeric targets across more than 16 food groups, with national adaptations (e.g., potatoes, oils and fats from foods, and dairy being optional via milk-calcium equivalents and beverage specifications). The alignment reveals very low consumption of legumes; limited consumption of vegetables, whole grains, and nuts/seeds (and fruit in men); and excess consumption of total and red/processed meat, ultra-processed foods (UPFs), free sugars/sugar-sweetened beverages, sodium, and alcohol. Biomarkers indicate a mean salt intake approximately two times the <5 g/day limit. Trans fatty acid (TFA) levels ≥ 0.5% persist in a substantial percentage of adults, predominantly from ruminant-derived TFAs. Sex-specific patterns are more adverse for men (e.g., meat, SSBs, alcohol, and sodium), whereas women have a higher intake of sweet UPFs. **Conclusions**: Slovenian diets are misaligned with the SNG2025. Priorities include increasing the intake of legumes, whole grains, vegetables, and nuts/seeds, while shifting protein sources away from red and processed meat. Additional priorities include reducing the intake of alcohol, sodium, free sugars, and UPFs through reformulation, procurement, and pricing/marketing measures, alongside routine biomarker and UPF surveillance. The SNG2025 enable monitoring and targeted implementation. Considering the limitations of the Si. Menu 2017/18 dataset, which includes food-group aggregation and limited information on food preparation, the results should be interpreted with caution with respect to the magnitude of deviations from SNG2025 targets, while the overall direction of misalignment remains robust.

## 1. Introduction

Slovenia has adopted the Slovenian Nutrition Guidelines 2025 (SNG2025), a quantitative, plant-forward framework aligned with the EAT–Lancet planetary diet and tailored to the national context. In accordance with the Ministry of Health, the SNG2025 include evidence-based recommendations for “Eating for Health and the Planet” and environmental sustainability, as well as public-facing materials such as “The Balanced Plate” that operationalise three plant-forward plate models—Mediterranean, lacto-ovo-vegetarian, and whole-food plant-based models—for healthy adults (≥18 y) [1,2,3,4]. A detailed description of the SNG2025 is provided elsewhere [4]. Conceptually, both the SNG2025 and the EAT–Lancet reference diet integrate health outcomes and environmental sustainability; limits on several food groups (e.g., red meat and animal fats) are set using health evidence and planetary boundaries [3]. In contrast, prior Slovenian food-based dietary guidelines (FBDGs) and legacy materials from the National Institute of Public Health of the Republic of Slovenia (NIJZ) (2000–2015) were largely qualitative. Key materials included the “*12 Steps to Healthy Eating*”, “the *Food Guide Pyramid*”, and “*Healthy Plate*” brochures [5,6,7]. Despite long-standing national advice, current Slovenian dietary intake remains misaligned with public health and planetary health objectives: population studies report underconsumption of legumes, whole grains, vegetables, fruits, nuts and seeds, dietary fibre, and folate and overconsumption of red/processed meat, salt, free sugars, refined grains, ultra-processed foods (UPFs), and alcohol [8,9,10,11,12,13]. National sodium exposure data corroborate excess salt intake [14].

From a health perspective, key risk factors remain highly prevalent (overweight/obesity, hypercholesterolaemia, and elevated blood pressure), and alcohol is an important, often underestimated risk factor because it is linked to cardiovascular disease (CVD), liver and gastrointestinal diseases, and several cancers [15,16,17]. From a health and environmental perspective, plant-forward dietary patterns are expected to deliver health benefits alongside environmental cobenefits, given the food system’s contribution to greenhouse gas emissions and resource use [18,19].

This paper has the following two objectives: (i) to provide a side-by-side, quantitative comparison of SNG2025, EAT–Lancet diet reference ranges, previous Slovenian FBDGs, and current Slovenian adult intakes; and (ii) to assess the sex-specific alignment of Slovenian adult intakes with SNG2025 using the Si. Menu 2017/18 [8] (Table 1 and Table 2). Health and environmental impact modelling, as well as the daily plate values used for such modelling, will be addressed in a companion manuscript.

The SNG2025 used in this comparative analysis represent the final, thoroughly reviewed version of the guidelines. Comments from all international and national reviewers have been incorporated. The completed SNG2025 were officially submitted to the Minister of Health on 3 October 2025, and are currently awaiting formal national approval. Accordingly, the guidelines are scientifically finalised but not yet formally adopted or implemented.

To date, FBDGs have not been quantitatively compared with international dietary benchmarks that jointly address health, environmental sustainability, and equity, and the dietary intake of Slovenian adults has not been assessed against the EAT–Lancet planetary health diet. Previous national analyses have focused primarily on nutrient adequacy or health-related outcomes, without integrating environmental sustainability and equity considerations into population-level intake assessments.

This study addresses this gap by operationalising the SNG2025 into quantitative targets and applying them to nationally representative dietary intake data. By jointly assessing alignment with reference frameworks that integrate health, environmental sustainability, and equity, this work provides an original, data-driven evaluation that extends beyond descriptive guideline reporting and enables a comparative, policy-relevant interpretation of population dietary patterns.

We hypothesise that the alignment of the observed dietary intake of Slovenian adults with the SNG2025 will reveal systematic misalignment with health-, environmental sustainability-, and equity-oriented targets, particularly for plant-based food groups and animal-sourced foods.

We further hypothesise that applying an integrated health–sustainability–equity framework (including the EAT–Lancet reference diet) will identify dietary gaps that were not fully captured by previous Slovenian FBDG assessments, which focused primarily on health-related outcomes.

## 2. Methods

The Methods section is structured to clearly distinguish between data sources (Section 2.1), harmonisation rules applied to enable comparability (Section 2.2), and analytical procedures used for the alignment assessment (Section 2.3).

### 2.1. Data Sources and Comparison Framework (SNG2025; EAT–Lancet Diet; Previous Slovenian FBDGs; and Si. Menu 2017/18)

We compared quantitative food group targets across four sources—SNG2025, the EAT–Lancet, both plant-forward reference diets [3], and previous Slovenian FBDGs—and observed Slovenian intakes from the Si. Menu 2017/18 [8]. For the previous Slovenian FBDGs, we used the “Zdrav krožnik” (Healthy Plate) brochure as the sole source that provides partial quantities; other NIJZ materials were qualitative [7]. For categories where the Healthy Plate brochure lacked numeric targets (e.g., cereals/grains; potatoes and other starchy foods; legumes/soy; and nuts and seeds), Table 1 indicates “not specified.” Observed intakes were obtained from the nationally representative Si. Menu 2017/18 survey (food group intake, sex-specific) [8]. Population trans fatty acid (TFA) shares were taken from the Si. Menu-based national analysis [20]. Sodium levels were corroborated using a 24-h urinary sodium study [14].

### 2.2. Harmonisation Rules

#### Standardisation and Units in SNG2025

All targets and intakes are reported per 2500 kcal/day as grams (g) or millilitres (mL) and refer to raw edible amounts unless marked “cooked”. We applied four harmonisation rules: milk-calcium equivalents (MCE), fat/oil counting, the World Health Organization (WHO)/Scientific Advisory Committee on Nutrition (SACN) limits for free sugars and salt, and the conversion of per-day display values to weekly caps (the weekly cap governs adherence).

Potatoes and starchy tubers. Target: ≤200 g/day cooked, boiled, and/or baked (not deep-fried) and without added saturated/trans fats; only small amounts of plant oil and iodised salt are advised.

Dairy/alternatives and MCE. Dairy is optional, with a default of 250 g/day, within an allowance of 0–500 g/day, expressed as MCE. We harmonised dairy and fortified alternatives using 1 MCE ≈ 300 mg Ca (and 0.4 MCE ≈ 120 mg Ca). Fortified plant drinks/yoghurts were counted as dairy only when fortified with calcium (and, conceptually, fortified with vitamin B12/vitamin D and iodine, as per the SNG2025). For ease of comparison in tables/figures, dairy intake was also reported “as milk” using the conversions provided in the Table 1 footnotes [21].

Oils and fats from foods. Target: ≤25 g of fats/day from whole foods (avocados and olives) and/or plant oils (olive, sunflower, rapeseed, corn/maize, pumpkinseed, flax/linseed, and walnut); nuts and seeds are counted separately at 30 g/day. The advice is to limit animal fats (butter, lard, and ghee) and tropical oils/fats (coconut and palm). For analysis, we operationalised this variable as a combined upper limit of 25 g/day for added plant oils, avocados, and olives (30 g/day). For household interpretation, we used 1 tbsp plant oil ≈ 10 g of fat (≈10 mL), ½ small avocado (≈50 g edible) ≈ 10 g of fat, and 16–20 pitted olives (≈45–60 g drained) ≈ 5 g of fat. Environmental guidance favours shifting from animal-based fats to locally/regionally produced plant oils and avoiding palm oil. Practical counting rules are provided under Table 1 (footnote d).

Free sugars and salt. The definitions and limit thresholds followed the WHO/SACN: free sugars ≤ 5% of total energy (the WHO also provides <10% as an upper bound) and salt < 5 g/day (approximately 2000 mg sodium); clarifying notes accompany Table 1 [22,23,24].

Weekly upper limits. The weekly upper limit for total meat (≤300 g/week) was reported as a daily equivalent of 43 g/day with an allowable day-to-day range of 0–86 g to aid in comparisons across rows. This approach was used as a convention for display only: 86 g/day × 7 is not permitted; the binding limit remains ≤300 g/week.

These harmonisation choices (e.g., milk-calcium equivalents, oil/fat equivalents, weekly-to-daily conversions, and standardisation to 2500 kcal/day) introduce uncertainty in the absolute magnitude of deviations from guideline targets. However, they are unlikely to affect the overall direction of misalignment observed across major food groups and are appropriate for the study’s objective of population-level, policy-oriented dietary benchmarking.

### 2.3. Analytical Procedures

#### 2.3.1. Food Group Mapping and Adherence Classification (Table 2)

Si. Menu 2017/18 foods were mapped to SNG2025 categories using ingredient composition and dominant component rules (mixed dishes were allocated to their principal constituents; tofu/tempeh/soy drinks were mapped under soy foods; and sugar-sweetened beverages (SSBs), water, and unsweetened tea were recorded separately where available). For each category, we computed the difference versus target and classified adherence as within (on target/within range), below (population mean below the lower bound), or above (population mean above the upper bound). Table 2 reports sex-specific adherence across 15 key food groups (see Section 2.2 for the standardisation rules).

#### 2.3.2. External Corroboration

Given underreporting in self-reports for sodium, primary estimates used 24-h urinary tests [14]. The Si. Menu-based national analysis was used to assess TFA exposure.

#### 2.3.3. Comparability Constraints

Direct comparisons to SNG2025 were not possible for several domains because of the limited granularity in Si. Menu 2017/18. Critically, Si. Menu does not capture either the amounts of whole grains or the quality of grain products (no distinction is made between white vs. whole-meal bread, refined vs. whole-grain pasta, or white vs. brown rice). Consequently, the whole grain quantity and adherence for cereals/grains cannot be computed, and the cereals/grains row is flagged as “no data”. Further limitations include the lack of dedicated variables for soy foods, herbs/spices, selected high-salt/high-fat subgroups, and the percentage of energy from saturated fat (SFA), and no details are available on the sweetening of tea/coffee/breakfast cereals or the type of coffee. The cooking method is not recorded, and, thus, some values reported for potato intake may include fried forms. Adherence is quantity-based only and should be interpreted in conjunction with the preparation standard in the notes of Table 1 (boiled/baked, not deep-fried).

Overall, the analytical framework applied in this study can be summarised as follows. The analytical approach was hypothesis-driven but noninferential by design. Although a priori research hypotheses were formulated, inferential statistical testing was not performed because the analysis relied on aggregated, sex-specific population means from national dietary surveillance data (Si. Menu 2017/18) and external biomarker sources. Consequently, alignment with SNG2025 and the EAT–Lancet reference diet was assessed using descriptive, aggregate-level metrics, including population means, percentages of guideline targets or limits, and classification of intakes as below, within, or above predefined thresholds. This approach is appropriate for quantitative guideline benchmarking and population-level policy monitoring rather than individual-level inference. All alignment assessments are based on sex-specific population means derived from nationally representative surveillance data; individual-level intake distributions and variance estimates were not available for the present analysis.

#### 2.3.4. Terminology

The term “plant-forward” refers to a predominantly plant-based approach that allows for modest amounts of dairy, eggs, fish, and meat. The term “*whole-food, plant-based (WFPB)*” refers to a vegan pattern that emphasises minimally processed plant foods and limits the use of animal products [25]. Concise definitions are provided here to ensure consistent mapping; expanded definitions appear in the companion manuscript.

#### 2.3.5. Comparative Specificity in SNG2025 (Bioavailability and Supplementation Guidance) Was Not Used as an Adjustment Factor

The SNG2025 provide explicit, user-level guidance that was absent in previous Slovenian FBDGs [7].

Iron bioavailability (nonhaem): Advice to drink black/green tea/coffee ≥ 1 h after meals and ≥2 h before or after iron supplements and to pair plant-based iron sources with vitamin C-rich foods [26,27,28,29].

Supplementation: Seasonal vitamin D at mid-latitudes; vitamin B12 is mandatory for WFPB (advised for vegetarians if fortification is inconsistent); EPA + DHA ≈ 250 mg/day from oily fish or algal/fish oil in plant-forward patterns and for pregnant and lactating women [30,31,32,33].

These instructions document the scope and were not applied to up- or downweight observed intakes in the alignment analysis.

## 3. Results

**Figure 1:**
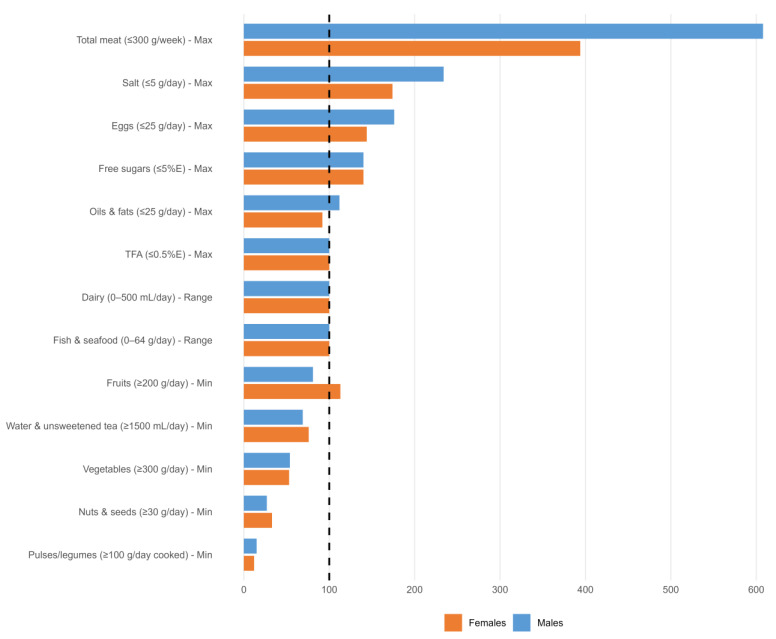
Alignment of intake in Slovenian adults (Si. Menu 2017/18) with the SNG2025 by sex: % of daily guidance (g/day, unless indicated otherwise; per 2500 kcal). Dashed line = 100% (within target). The percentage (%) was computed as intake/target × 100 (≥targets) or intake/limit × 100 (≤limits). These percentages indicate relative alignment with the SNG2025 targets or limits and are intended for operational comparison within a policy framework, not as absolute or clinical measures of dietary adequacy; as relative metrics, they may visually accentuate deviations and should be interpreted with caution regarding the exact magnitude of departures from the targets. Range items (fish/seafood, 0–64 g/day and dairy, “as milk” 0–500 mL/day) shown at 100% for both are within; midpoints are reported in the text. Total upper limit of meat intake: ≤300 g/week. M = blue and F = pink. Status legend (M/F): above ≥ upper limit; within = within target; and below ≤ target. Items not plotted: SSBs, above/above; cocoa/hot chocolate, above/above; wine M:F ≈ 2:1, above/above; beer, M:F ≈ 4:1, above/above; spirits, M:F ≈ 4:1, above/above; UPFs, above/above; and butter and other animal fats, above/above. Abbreviations: M = males, F = females, SSBs = sugar-sweetened beverages, and UPFs = ultra-processed foods.

Overview. Table 1 compares the SNG2025 with the EAT–Lancet diet [3] as well as the previous Slovenian FBDGs [7] and nationally representative intakes from Si. Menu 2017/18 [8,20] (including Si. Menu-based TFAs with 24-h urinary sodium as a biomarker of corroboration [14]. Values are reported in g/day (or mL/day) and represent raw edible amounts, unless indicated otherwise. Notes under Table 1 indicate harmonisation rules (MCE and WHO limits). Table 2 reports sex-specific alignment with the SNG2025 across 15 food groups 2017/18. Figure 1 shows sex-specific adherence across key food groups as “% of SNG2025 daily guidance,” with 100% indicating “within”. Daily plate values (Mediterranean, vegetarian, and WFPB) are detailed in the companion manuscript [4].

### 3.1. Quantitative Comparison: The SNG2025 vs. EAT–Lancet Diet vs. Previous Slovenian FBDGs (Table 1)

Quantification gap. The SNG2025 and the EAT–Lancet diet provide gram-level targets for nearly all food groups. In contrast, prior Slovenian FBDGs were largely qualitative (e.g., no numeric targets for grains, potatoes, legumes/soy, meat/fish, nuts/seeds, or oils/fats), limiting historical comparability.

Key quantitative differences across frameworks are described below.

Staples. The SNG2025 set a limit of ≤200 g/day (cooked) potatoes, which is higher than that in the EAT–Lancet diet (≈50 g/day, range 0–100 g/day). Both frameworks emphasise whole grains, pulses, and soy foods, but these aspects were not quantifiable in the prior Slovenian FBDGs because of the lack of numeric targets.

Animal-sourced foods (optional). According to the SNG2025 and EAT–Lancet diet, animal-sourced foods are optional (which was not previously the case). Milk and dairy are reframed from 400–600 mL/day (previous Slovenian FBDGs) to ~250 mL/day within a 0–500 mL/day range, expressed as MCE, allowing substitution with fortified plant alternatives when preferred.

Fats/oils. The SNG2025 apply a combined upper limit of ≤25 g/day for added oils, avocados, and olives (nuts/seeds counted separately). The EAT–Lancet Commission dietary guidelines use separate limits, allowing up to ~40 g/day of unsaturated plant oils and restricting palm/animal fats. Prior Slovenian materials provided qualitative information on fat types but no clear quantitative limits.

Beverages. The SNG2025 include explicit guidelines (water, mineral water, and unsweetened tea are defaults; limits for coffee and fruit juice; and avoidance of SSBs, especially energy drinks). This level of specificity is absent in the EAT–Lancet diet and in the prior Slovenian FBDGs.

Alcohol. The SNG2025 recommend complete abstinence, aligning with the WHO/WCRF recommendations. Previous Slovenian FBDGs permitted moderate consumption (≤20 g/day for men and ≤10 g/day for women).

UPFs. Both the SNG2025 and the previous Slovenian FBDGs recommend avoiding or minimising UPFs. The scope and operational details of UPF-related guidance differ across frameworks.

Salt. The SNG2025 and previous Slovenian FBDGs specify <5 g salt/day (≈<2 g sodium/day), which is consistent with the WHO; EAT–Lancet emphasises reducing UPFs (primary sodium source).

Free/added sugars. The SNG2025 and EAT–Lancet diet limit free sugars to <5% energy intake (≈≤31 g at 2500 kcal; explicitly listed in the EAT–Lancet diet), aligning with the SACN (≤5% energy intake) and consistent with the WHO’s conditional recommendation for <5% energy intake (stricter than the WHO’s strong recommendation for <10% energy intake). Previous Slovenian FBDGs allowed 5–10% energy intake from added sugars.

### 3.2. Alignment of Slovenian Intakes with SNG2025 (Table 2)

#### 3.2.1. Adherence to Recommended Intakes

Range-type guidance is, by definition, “within” when intakes fall anywhere inside the band. Dairy “as milk” (0–500 mL/day) and fish/seafood (0–64 g/day) are, thus, coded as within; for context only (not used for ranking), mean dairy consumption ≈ 292 mL (~117% of a 250 mL reference) and fish consumption vs. a 32 g midpoint equals 84% (men)/56% (women). Females’ added fats/oils (~23 g/day) are within the ≤25 g/day limit, and fruit meets the 200 g/day target (~226 g/day).

The average milk and dairy intake was ≈292 mL/day in both sexes, with ≈14% non-consumers, indicating alignment with the optional SNG2025 allowance (0–500 mL/day). Fish and seafood met the weekly flexibility band for both sexes (men ≈ 27 g/day and women ≈ 18 g/day). Added fats and oils were within the ≤25 g/day limit for women (≈23 g/day). Fruit consumption was ≥200 g/day for women (approximately 226 g/day). Midpoint comparisons are provided only for context: dairy ≈ 117% of a midpoint 250 mL/day reference and fish compared to a 32 g/day midpoint: 84% (men)/56% (women).

#### 3.2.2. Inadequate Intake

The intake of several plant-based groups remained well below the targets. Vegetable intake averaged ≈160 g/day in both sexes—approximately 54% (men)/53% (women) of the ≥300 g/day target. Fruit intake was below the 200 g/day target in men at ≈162 g/day (81% of the target) but on target in women. The intake of legumes (excluding soy) was particularly low (men, 14.6 g/day and women, 11.6 g/day), corresponding to approximately 15%/12% of a ≈100 g/day (cooked) benchmark. The intake of nuts and seeds reached only approximately 27% (men)/33% (women) of the ≥30 g/day recommendation (≈8–10 g/day). The intake of water and non-alcoholic beverages fell short of ≥1500 mL/day, especially in men (drinking water ≈ 925 mL/day and tea ≈ 106 mL/day; women ≈ 970 mL/day and 172 mL/day, respectively; sweetening of tea not specified). The largest shortfalls (share of target) were observed for legumes at 15%/12%, nuts and seeds at 27%/33%, vegetables at 54%/53%, fruit at 81% (men), and water and non-alcoholic beverages at 69%/66%.

#### 3.2.3. Excessive Intake

The total amount of meat (meat and meat products) consumed substantially exceeded the ≤300 g/week limit: approximately 1825 g/week for men (608%) and approximately 1183 g/week for women (394%). In contrast to the advice to prioritise poultry over red meat in the SNG2025, men consumed ~2× more red meat than poultry, while women consumed similar amounts of red meat and poultry; processed meat intake was high in both sexes (especially men). Egg consumption exceeded the ≤25 g/day limit (44 g/day for men and 36 g/day for women; 176%/144%). The intake of added fats and oils was above the upper level in men (28 g/day = 112%). Free sugar consumption averaged 7% E in both sexes (~140% of the <5% E target), with SSBs contributing meaningfully (≈136 mL/day for men and 63 mL/day for women) [22].

Alcohol consumption was nonzero; compared with women, men reported consuming ≈4× more beer/spirits and ≈2× more wine. UPF intake (e.g., sweet bakery products and confectionery) was high in both sexes (sweet intake was higher in women). Salt, which is based on 24-h urinary sodium levels (2022), was 11.7 g/day in men (234%) and 8.7 g/day in women (174%), with a mean 10.3 g/day in 2022 vs. 11.3 g/day in 2010, i.e., still approximately 2× the WHO upper level [14,24,34]. TFA intake averaged ~0.38–0.50% E, yet ~29% of adults consumed ≥0.5% E [20]. The greatest percentages of overshoots (share of limit) were 608%/394% for total meat, 176%/144% for eggs, 112%/92% for added fats and oils, 140%/140% for free sugars, and 234%/174% for salt.

#### 3.2.4. Non-Consumers

The share declared as non-consumers was low for fresh meat (~1–3% overall) but higher for processed meat (11.8%) and fish (8.8%). Many adults reported no intake of eggs (41.2%), milk (13.5%), or cheese (5.5%); among plant foods, non-consumers accounted for 33.5% of the total respondents for nuts/seeds and 4.9% for legumes. For beverages/ready meals, 17.0% of the total respondents were non-consumers of alcoholic beverages overall (wine 26.4%, beer 31.6%, and spirits 92.9%), 31.3% were non-consumers of SSBs, and 34.6% were non-consumers of ready-to-eat meals [8].

#### 3.2.5. Sex-Specific Perspective

Compared with women, men showed a more adverse pattern overall, consuming more red/processed meat, eggs, added fats/oils, SSBs, alcohol, and sodium, whereas women showed greater exposure to sweet UPFs; both sexes share shortfalls in the intake of legumes, vegetables, and nuts/seeds. Accordingly, the priorities for men are a shift in protein consumption away from red/processed meat with concurrent reductions in the consumption of alcohol, SSBs, sodium, and added fats/oils; the priorities for women are a decrease in the consumption of sweet UPFs and attention to fish adequacy; and priorities for both sexes include substantial increases in the consumption of legumes, vegetables, and nuts/seeds to align with the SNG2025.

#### 3.2.6. Comparisons Constrained by Data Availability

Direct comparisons to the SNG2025 were not possible for whole-grain quality, soy foods (tofu/tempeh/soy drinks), herbs and spices, selected high-salt/high-fat product subgroups, and % E from SFAs due to missing or insufficient Si. Menu variables, including tea sweetening and coffee type, which were also unspecified.

**Table 1:** Quantitative comparison of the SNG2025 daily intake guidance (per 2500 kcal; g/day, unless indicated otherwise), EAT–Lancet ranges, previous Slovenian FBDGs, and intake by Slovenian adults (Si. Menu 2017/18, 18–64 y).

Food Group	SNG2025 Daily Intake Guidance (g/Day Unless Noted) ^a^ per 2500 kcal	EAT–Lancet Reference Intake (Possible Range), (g/Day Unless Noted) ^a^ per 2500 kcal	Previous Slovenian FBDG Daily Intake Guidance (g/Day Unless Noted) kcal Not Specified	Slovenian Dietary Intakes Mean (g/day, Unless Indicated Otherwise) Males/Females (18–64 Years)
Cereals/grains	≥230 g dry (≈600 g cooked grains), (≥50% whole-grain products, <50% refined grains)	232 g (125–232 g)	Not specified	Whole grains and whole-grain products: no data ^k^ Bread and bakery products: 210 g/145 g Breakfast cereals: 34 g/39 g Pasta, rice: 63 g/55 g
Potatoes and other starchy tubers	≤200 g cooked, boiled/baked; not deep-fried. Avoid butter/lard/margarine; add only small amounts of oil; use iodised salt sparingly.	50 g (0–100 g)	Not specified	Potatoes: 99 g/76 g
Vegetables	≥300 g	300 g (200–600 g) dark green ≈ 100 g red/orange ≈ 100 g other ≈ 100 g	250–400 g green, white, orange yellow, red, and blue violet	Total:163 g/158 g Fresh:122 g/125 g Preserved and canned: 39 g/30 g
Fruits	200 g (100–300 g)	200 g (100–300 g)	150–250 g	Total: 162 g/226 g Fresh: 141 g/198 g Canned, dry: 20 g/22 g
Pulses/legumes (dry beans, lentils, and peas) Soy foods (tofu/tempeh etc.)	≥50 g dry legumes (≈100 g cooked legumes) + ≥25 g dry soy foods (≈70 g cooked soybeans or tofu/tempeh (as soy equivalents))	50 g (0–100 g) dry beans, lentils, and peas + 25 g (0–50 g) soy foods	Encouraged as meat substitutes; no gram target specified	Legumes and legume products: 14.6 g/11.6 g cooked Soy foods: no data ^k^
Dairy or fortified plant-based alternatives ^b^ (1 MCE/day) ^c^	250 (0–500) mL milk/dairy (“as milk”) or calcium-fortified, plant-based drink/yoghurt ^b,c^	250 (0–500) dairy (milk or equivalent, e.g., cheese)	400–600 mL/day of part skim or low-fat milk or yoghurt: or ≈ 120–180 g soft cheese ≈ 60–90 g hard cheese	Milk: 80 g/84 g Yoghurt and cream: 72 g/95 g Cheese: 35 g/32 g “As milk”: 292 mL/292 mL ^c1^
Meat and processed meat ^d^	Total: 43 g/day, ≈≤300 g/week; (day-to-day 0–86 g/day; not 86 g × 7) ^d^ Prioritise poultry, reduce red and processed meat	29 g (0–58): poultry 7 g (0–14): beef and lamb 7 g (0–14): pork 0 (aim for none): processed meat Total: ≤300 g/week	No gram target specified. 1–3×/week: poultry (lean cuts, skin removed) ≤1×/week: red meat (beef, pork, lamb, and game; limited/occasional only; lean cuts, remove visible fat) 1–2 meat-free days/week, replace fatty meats/products with legumes, fish, poultry or lean meat, choose lean cuts and avoid processed meats	Poultry: 72 g/65 g Red meat: 137 g/76 g Red meat and poultry: 209 g/141 g Processed meat: 52 g/28 g Total: 261 g/169 g Total: 1825 g/week/1183 g/week
Fish and seafood	200 g/week (0–450 g) ≈29 g/day (0–64 g)	28 g (0–100 g)	No gram target specified. 1–2×/week (include fatty marine fish; baked/steamed, not fried)	Fish and fish products: 27 g/18 g
Eggs	≤25 g/day ≈3 eggs/week	13 g (0–25 g) ≈1–3 eggs/week	Not specified	Eggs, including from foods: 44 g/36 g
Nuts and seeds	≥30 g/day	50 g (25–75 g) 25 g (0–75) peanuts 25 g tree nuts	Not specified	Fresh and processed nuts and seeds: 8 g/10 g
Oils and fats from whole foods (target: ≤25 g/day in addition to nuts and seeds) ^e^	≤25 g fat/oil; or ≤100 g avocado (20 g oil) + 8–10 olives (5 g oil) ^d^ limit/avoid: lard/tallow, butter, cream, and tropical fat	Added fats: ^a,f^ 40 g unsaturated plant oils/day (20–80) 6.8 (0–6.8): palm oil 5 (0–5): lard/tallow 0: dairy fat (included within dairy)	Upper limits were not specified for olive, rapeseed/canola, sunflower, soybean, and corn oils limit/avoid: lard/tallow, palm/coconut fat, butter, cream; fatty/processed meats/cheeses	Total: 28 g/23 g Vegetable oils and margarines: 20 g/16 g Butter and other animal fats: 8 g/7 g
Herbs and spices	Use regularly to minimise salt/sugar intake	Not specified	Use fresh, dried, or frozen herbs and spices instead of salt	No data ^k^
Water and non-alcoholic beverages	≈1500 mL (mineral) water, unsweetened tea as the default, Coffee permitted (≤400 mg caffeine/day) Fruit juice occasionally, ≤200 mL/day Avoid SSBs, especially energy drinks	Not quantified; water as the default	≈1500–3000 mL (mineral) water), unsweetened fruit/herbal teas as the default fresh fruit/vegetable juices may be diluted 1:1 with water (consumed with meals)	Water: 925 mL/970 mL Tea: 106 mL/172 mL Coffee: 91 mL/104 mL Fruit/vegetable juices: 79 mL/46 mL SSBs: 136 mL/63 mL Cocoa and hot chocolate drink: 46 mL/46 mL
Alcohol	0 mL	No numeric recommendation	M ≤20 g alcohol/day (≤14 units/week; ≤5 units/occasion) F ≤10 g alcohol/day (≤7 units/week; ≤3 units/occasion). 1 unit = 10 g alcohol ≈100 mL wine, 250 mL beer, or 30 mL spirits	Alcoholic beverages (mL/day) Wine: 33/18 Beer: 183/44 Spirits: 17/4
Ultra-processed foods (UPFs) ^f^	Avoid or minimise (limits in the salt, sugars, and fat rows)	Not quantified; keep low; see sugar/fat limits	Avoid or minimise	High-sugar foods (sugar, confectionery, cakes, cookies, and desserts): 98 g/107 g High salt and fat foods: no data ^k^
Overall diet orientation ^i,j^	<10% E from SFAs; <5% E from added/free sugars ^g^ <0.5% E TFAs; <5 g/day salt ^h^	≤31 g/day (0–31 g) added/free sugars ^g^	Not specified. 5–10% E from added sugars avoid TFAs ≤5 g/day salt g	% E from SFAs: no data ^k^ 7%/7% E from free sugars 0.4–0.5% E from TFAs 11.7 g/8.7 g salt ^i^

^a^ Units/weights. Values are reported per 2500 kcal, amounts g/day (or mL/day) and refer to raw edible amounts unless marked “cooked.” ^b^ Fortification. For plant alternatives, unsweetened, calcium-fortified products are used; vitamin B12 and vitamin D/iodine fortification vary by brand (check labels) [35,36,37,38]. ^c^ Dairy allowance and milk-calcium equivalent (MCE). Dairy is optional in the SNG2025; the default is 250 g/day within 0–500 g/day, and reported as MCE. A total of 1 MCE (≈300 mg Ca) can be obtained from any one of the following: 250 mL of milk; 250 g of a calcium-fortified plant drink; 250 g of soy yoghurt; ≈50–75 g of soft cheese (e.g., camembert or brie); or ≈27–42 g of hard/semihard cheese (e.g., parmesan ≈ 27 g or cheddar/aged Tolminc ≈ 42 g). A total of 0.4 MCE (≈120 mg Ca) can be obtained from any one of the following: 100 mL of milk; 100 g of yoghurt; 100 mL of calcium-fortified plant drink; 100 g of soy yoghurt; ≈20–30 g of soft cheese; or ≈11–17 g of hard/semihard cheese (e.g., Parmesan ≈11 g or cheddar/aged Tolminc ≈ 17 g). Prefer reduced-fat and fermented dairy; consider the environmental impact: keep cheese portions modest relative to milk/yoghurt. Units: mL for beverages and g for foods/yoghurts [21]. ^c1^ “As milk”. For comparison with the 250 mL milk reference, yoghurt and cheese were converted to an equivalent milk volume (100 mL yoghurt ≈ 100 mL milk and 20 g cheese ≈ 100 mL milk). In Si. Menu, the “yoghurt and cream” category was split 50:50 (only the yoghurt half counted). M: 80 mL (milk) + 73 mL × 0.5 (yoghurt) + 35 g × (100/20) (cheese) = 292 mL (≈117% of 250 mL). F: 84 mL (milk) + 96 mL × 0.5 (yoghurt) + 32 g × (100/20) (cheese) = 292 mL (≈117%). ^d^ Total meat (weekly upper limit). Forty-three grams per day is the per-day equivalent of ≤300 g/week. Day-to-day 0–86 g; weekly upper limit ≤300 g; and do not interpret 86 g/day × 7 as permitted. ^e^ Oils and fats. Daily limit: ≤25 g from plant oils and whole-food fat sources (avocados or olives). Nuts/seeds are counted separately: 30 g/day (not counted in the 25 g). How to count: 1 tbsp plant oil ≈10 g (≈10 mL); ½ small avocado (≈50 g edible) ≈10 g fat; and 16–20 pitted olives (≈45–60 g drained) ≈ 5 g fat.) Examples: (i) 2½ tbsp oil (≈25 g); (ii) 1 tbsp oil (≈10 g) + ½ small avocado (≈10 g) + 6–10 olives (≈5 g); and (iii) 1½ tbsp oil (≈15 g) + ½ small avocado (≈10 g). Preference: Choose nontropical plant oils (olive, rapeseed, sunflower, maize/corn, pumpkin seed, flax/linseed, and walnut). Limit animal fats and avoid palm/coconut oil/fat. ^f^ UPFs. Industrial formulations with cosmetic processing (e.g., SSBs, confectionery, refined-flour snacks, and processed meats) that are typically high in salt, free sugars, and SFAs/TFAs: avoid/minimise and see salt/sugar/fat rows for limits; practical tips in [4]: Supplementary Box S1. ^g^ Free sugars. WHO definition (adding sugars plus sugars in honey, syrups, fruit juices, and juice concentrates) [23]; target <5% of total energy [22]. ^h^ Salt. The WHO adult limit is <2000 mg sodium/day (≈<5 g salt/day) [24]. ^i^ Salt (biomarker). Salt intake is estimated from 24-h urinary sodium intake using 17.1 mmol Na ≈ 1 g salt (NaCl); NIJZ 24-h sodium study 2022/23) [14]. ^j^ Supplementation (model assumption). Seasonal vitamin D; vitamin B12 mandatory for WFPB (advised for vegetarians if fortification is inconsistent); and EPA + DHA ≈250 mg/day from oily fish or algal/fish oil. ^k^ Comparability constraints in Si. Menu. Several domains are not directly comparable because of limited variable granularity: (i) no dedicated fields for soy foods, selected high-salt/high-fat subgroups, caffeine, or % energy from SFAs; (ii) cereals/grains are not distinguished as whole-grain vs. refined, and, thus, adherence cannot be computed; (iii) yoghurt is reported together with cream, and, thus, yoghurt cannot be isolated; (iv) cheese is not split into soft vs. hard, and MCE comparisons use total cheese and should be interpreted with caution; and (v) cooking methods for potatoes are not captured in Si. Menu, and values may include fried forms, where the SNG2025 require boiling/baking. Data sources: EAT–Lancet diet reference ranges [3]; previous Slovenian FBDGs [7]; and nationally representative intakes from Si. Menu 2017/18 [8], including the TFAs from the Si. Menu-based analysis [20] and biomarker sodium levels from the 24-h urinary sodium study [14]. Abbreviations: DHA, docosahexaenoic acid; % E, percentage of total energy; EPA, eicosapentaenoic acid; F, females; M, males; MCE, milk-calcium equivalent; SFA, saturated fatty acids; SSBs, sugar-sweetened beverages; TFAs, trans fatty acids; UPFs, ultra-processed foods; and WFPB, whole-food, plant-based.

**Table 2:** Alignment of intake by Slovenian adults (Si. Menu 2017/18) with the SNG2025 for 15 food groups. The values are presented as sex-specific means; the last column shows the percentage of target/limit (100% = within).

Food Group	SNG2025 (Mean of 3 Plates) Daily Intake Guidance (g/Day Unless Noted) per 2500 kcal ^a^	Slovenian Dietary Intakes Mean (g/Day, Unless Indicated Otherwise) Males/Females (18–64 Years)	Below/Within/Above % of Target Males/Females
Cereals/grains	≥230 g dry (≈600 g cooked), quality: ≥50% whole grains	No indicator for whole-grain vs. refined products	Whole-grain quality missing: Not computable
Potatoes and other starchy tubers	≤200 g cooked ≤200 g cooked, boiled/baked; not deep-fried. Avoid butter/lard/margarine; add only small amounts of oil; use iodised salt sparingly.	Potatoes: 99 g/76 g (no data for other starchy tubers)	Within/Within 50%/38% of limit (≤200 g cooked/day)
Vegetables	≥300 g	Total: 163 g/158 g	Below/Below 54%/53%
Fruits	200 g (100–300 g)	Total:162 g/226 g	Below/Within 81%/113%
Pulses/legumes (dry beans, lentils, and peas) Soy foods (tofu/tempeh, etc.)	≥50 g of dry legumes (≈100 g of cooked legumes) + ≥25 g of dry soy foods (≈70 g of cooked soybeans or tofu/tempeh (as soy equivalents))	Legumes and legume products: 14.6 g/11.6 g cooked Soy foods: No data	Below/Below 15%/12% Soy foods: Not computable
Dairy or fortified plant-based alternatives ^b^ 1 MCE (~300 mg Ca)/day ^d^	250 (0–500) mL “as milk” or yoghurt/day = 250 (0–500) mL of fortified, plant-based drink/yoghurt; or ≈50–75 g of soft cheese or ≈27–42 g of hard cheese	Milk: 80/84 g Yoghurt and cream: 73 g/96 g Cheese: 35 g/32 g No separate data 292/292 mL “as milk” ^c1^	Within/Within 100%/100% (117%/117% vs. 250 mL/day midpoint)
Meat and processed meat	Total: 43 g/day (0–86 g) ≤300 g/week Prioritise poultry, reduce red meat and processed meat	Total: 261 g/169 g 1825 g/week/1183 g/week Poultry: 72 g/65 g Red meat: 137 g/76 g Red meat and poultry: 209 g/141 g Processed meat: 52 g/28 g	Above/Above Total: 608%/394%; (6.1×)/(3.9×) the limit Red meat vs. poultry: (2× >/similar) Processed meat
Fish and seafood	200 g/week (0–450 g) ≈29 g/day (0–64 g)	Fish and fish products: 27 g/18 g	Within/Within 100%/100% ^b^ (84%/56% vs. 32 g/day midpoint)
Eggs	≤25 g/day ≈3 eggs/week	Eggs, including from foods: 44 g/36 g	Above/Above 176%/144%
Nuts and seeds	≥30 g/day	Nuts and seeds: 8 g/10 g	Below/Below 27%/33%
Oils and fats from whole foods (target: ≤25 g/day in addition to nuts and seeds) ^d^	≤25 g of oil; or ≤100 g of avocado + 8–10 olives; or ≤20 g of oil + 8–10 olives limit/avoid: lard/tallow, tropical fats, butter, and cream	Total: 28 g/23 g Vegetable oils and margarines: 20 g/16 g Butter and other animal fats: 8 g/7 g	Above/Within of limit (≤25 g/day) 112%/92% Above/Above
Herbs and spices	Use regularly to minimise salt/sugar intake	No data	Not computable
Water and non-alcoholic beverages	≈1500 mL (mineral) water, unsweetened fruit/herbal teas are the default, Coffee permitted (≤400 mg caffeine/day) Fruit juice occasionally, ≤200 mL/day Avoid SSBs, especially energy drinks	≈1031 mL/1142 mL of water, tea Water: 925 mL/day/970 mL/day Tea: 106 mL/172 mL Coffee: 91 mL/104 mL Fruit/vegetable juices: 79 mL/46 mL SSBs: 136 mL/63 mL Cocoa and hot chocolate drink: 46 mL/46 mL	Below/Below 69%/76% Caffeine: Not computable Juices: Within/Within SSBs: Above/Above Cocoa and hot choc. drink: Above/Above
Alcohol	0 mL	Alcoholic beverages (mL/day) Wine: 33 mL/18 mL Beer: 183 mL/44 mL Spirits: 17 mL/4 mL	Above/Above Wine: ≈2× in M vs. F Beer: ≈4× in M vs. F Spirits: ≈4× in M vs. F
Ultra-processed foods (UPFs) ^f^	Avoid or minimise (see limits in the salt, sugar, and fat rows)	High-sugar foods (sugar, confectionery, cakes, cookies, and desserts): 98 g/107 g High-salt and high-fat foods: no data	Above/Above High-salt and high-fat foods: Not computable
Overall diet orientation ^i, j^	<10% E from SFAs <5% E from free sugars ^g^ <0.5% E from TFAs <5 g/day salt ^h^	% E from SFAs: No data 7%/7% E from free sugars 0.4–0.5% E from TFAs 11.7 g/8.7 g salt	% E from SFAs: Not computable ^k^ Above/Above 140%/140% free sugars Within/Within ≈100%/≈100% TFA Above/Above 234%/174% salt

^a^ Units/scope. The SNG2025 guidance is per 2500 kcal and Si. Menu values are sex-specific means for adults aged 18–64 years. ^b^ Mapping and adherence. Si. Menu items were mapped to SNG2025 food groups. Within/below/above are based on the population mean vs. the SNG2025. Weekly upper limits (e.g., ≤300 g/week of total meat and ≤3 eggs/week ≈≤25 g/day) are shown as per-day equivalents for display but interpreted weekly in the text. For ≥targets: % = intake/target × 100 (≥100% = within and <100% = below). For ≤limits: % = intake/limit × 100 (<100% = within; 100% = at limit; and >100% = above). Range targets (e.g., fish, 0–64 g/day and dairy, 0–500 mL/day) were counted as within. ^d^ Dairy/MCE. Intakes were harmonised using MCE; see Table 1; note ^c^ for conversions [21]. Not computable: The Si. Menu variable was missing. Cross-references: Table 1—fortification (note ^b^); “as milk” (note ^c1^); UPFs (note ^f^); free sugars (note ^g^); salt (note ^h^); salt biomarker (note ^i^); supplementation (note ^j^), and comparability constraints in Si. Menu (note ^k^). Data sources. See Table 1. Abbreviations. F, females; M, males; % E, percentage of energy; MCE, milk-calcium equivalent; SSBs, sugar-sweetened beverages; TFA, trans-fatty acids; and UPFs, ultra-processed foods.

## 4. Discussion

As shown in the Results section, the SNG2025 translate a plant-forward paradigm into quantified results that are aligned with the EAT–Lancet reference diet and adapted to the national context [3]. The discussion, therefore, focuses on the interpretative implications of this framework, rather than reiterating numerical comparisons.

### 4.1. Quantitative Comparison with the EAT–Lancet Diet and Previous Slovenian FBDGs (Table 1)

Compared with the previous Slovenian FBDGs, the SNG2025 represent a methodological advance by providing gram-level targets across food groups that were previously addressed only qualitatively [7]. This shift enables baseline-to-target monitoring and clearer identification of priority gaps, particularly for protective plant-based foods.

Several domains central to population misalignment are addressed more explicitly than before, including the optional role of animal-sourced foods, flexible dairy allowances enabling fortified plant alternatives, and clearer guidance on beverages, free sugars, and alcohol. In addition, harmonised counting rules (e.g., milk equivalents and oil equivalents) translate nutrient intent into comparable food-based metrics, thereby improving interpretability for surveillance and counselling.

Taken together, the SNG2025 offer a more coherent and measurable framework than the previous Slovenian FBDGs by integrating health, environmental sustainability, and equity in a single, measurable package aligned with international reference diets [3,19,39].

### 4.2. Alignment of Slovenian Intakes with the SNG2025 (Table 2)

Overall, dietary intake in Slovenia shows consistent shortfalls in protective plant-based foods alongside excesses in red/processed meat, free sugars (especially SSBs), salt, and alcohol. Range-based items (such as fish and dairy) generally fall within their permitted limits and do not contribute to misalignment. Because very few adults are true non-consumers of meat, risk reduction must target habitual consumers—particularly men—while supporting adequacy among those who avoid milk or fish through fortified alternatives. After the reformulation, industrial TFAs are low on average, but residual ruminant TFAs from butter/meat justify continued surveillance. Sodium remains the dominant component consumed in excess based on biomarker data, despite a slight improvement since 2010, highlighting persistent structural contributors to the food supply and out-of-home eating.

These patterns underscore that the main contributors to misalignment with the SNG2025 are red/processed meat, salt, and free sugars, especially SSBs and alcohol, while the consumption of legumes, vegetables, and nuts/seeds remains insufficient. The prominence of beverages within free sugars and alcohol intake, especially among men, suggests that this domain disproportionately contributes to observed excesses, which is consistent with the findings of previous Slovenian intake analyses [8,14,20,24,40,41].

Dairy is optional in the SNG2025, with a flexible MCE allowance. Milk and dairy are primary dietary sources of SFAs [42], while the environmental footprint of cheese is greater than that of milk/yoghurt, and both exceed that of plant-based alternatives. Approximately 14% of adults report no milk intake [8], underscoring the role of appropriately fortified alternatives (calcium, vitamin B12, vitamin D, and iodine) and routine B12 guidance in plant-forward patterns. The SNG2025 recognise unsweetened, calcium-fortified soy drinks as the closest nutritional substitutes because of their comparable protein content; other plant-based drinks are not nutritionally equivalent unless they are fortified. Unfortified alternatives may increase the risk of long-term inadequacy in calcium, vitamin B12, and protein [3,37,38,42,43,44,45]. TFAs and sodium are surveillance priorities. Mean industrial TFA exposure is low after the reformulation, but ruminant TFAs (butter/meat) maintain a nontrivial tail above 0.5% E, justifying continued label/product surveillance [20]. Sodium remains the dominant nutrient consumed in excess on a biomarker basis, despite slight improvements since 2010, indicating that reformulation efforts should continue to prioritise staple foods, processed products, and out-of-home meals [17,24]. Overall, comparison with the SNG2025 indicates substantial directional gaps relative to the benchmark, particularly for legumes, vegetables, nuts and seeds, and fruit (in men)—and for several “limit” components, including total and red/processed meat, free sugars/SSBs, sodium, alcohol, and added fats. These patterns are summarised in Figure 1 and Table 2.

### 4.3. Relation to Earlier Si. Menu Interpretations

Our findings reinforce and extend the prior Si. Menu analyses but differ in terms of the classification because the comparator has changed. Previous Si. Menu interpretations primarily referenced previous German DGE cut-offs (Nutrition Circle) [46], whereas we assess alignment against the SNG2025, which explicitly integrate health, environmental sustainability, and equity. Consequently, some categories previously judged as “adequate” are reclassified as being below/above the target under the SNG2025, reflecting a stricter and system-aligned benchmark rather than a deterioration in dietary quality per se.

The present analysis should be interpreted within the context of a newly adopted policy framework. The observed degree of misalignment reflects alignment with the SNG2025 as a normative benchmark that integrates health and environmental sustainability considerations rather than an absolute measure of dietary inadequacy per se. Consequently, part of the observed gap—particularly regarding the balance between animal- and plant-based foods—represents a redefinition of dietary adequacy within a more plant-forward, sustainability-oriented framework rather than a deterioration in nutritional quality compared with previous guidelines. Similar patterns of misalignment between current population intakes and newly adopted healthy and sustainable dietary guidelines, which are characterised by high intakes of animal-sourced foods and insufficient intakes of plant-based foods, have been reported in multiple European countries and other countries, including the Nordic countries [47], Denmark [48], Germany [49], Austria [50], and Canada [51]. In parallel, excessive intake of SSBs, free sugars, salt, and UPFs is consistently reported across European populations and globally [52,53,54,55].

### 4.4. Dietary Gaps and the Health Burden in Slovenia

Despite the availability of previous Slovenian FBDGs and international targets, prevailing adult diets in Slovenia remain misaligned with public health and planetary health objectives. Population surveys document the underconsumption of vegetables, fruits, whole grains, legumes, nuts, and seeds and overconsumption of red/processed meat, UPFs, free sugars (mostly from SSBs), SFAs/TFAs, salt, and refined grains [8,9,56]. Comparable directional gaps have also been reported among Slovenian adolescents, including low fibre and fruit/vegetable intake and high intake of meat/meat products, free sugars (especially SSBs), and sodium [57,58]. These consistencies suggest system-level drivers (UPF availability, product formulation, and marketing/placement). We did not perform an adolescent alignment against SNG2025 here, as the methods and targets differ and warrant a dedicated analysis. These patterns co-occur with a high burden of overnutrition and cardiometabolic risk—approximately 59% of adults are overweight (≈74% ≥65 years), 56% have elevated blood cholesterol levels, and 48% have high blood pressure [2,8,11,16,41,56,59]. Alcohol remains a major risk factor with broad disease links (cardiovascular, hepatic, and gastrointestinal diseases, as well as multiple cancers) and imposes substantial social and health care costs in Slovenia. For 2012–2016, total alcohol-related costs were approximately EUR 228 million per year, which corresponds to ≈approximately 0.6–0.7% of the GDP during that period [15,16,60,61,62]. Given the absence of a safe level of alcohol consumption with respect to cancer risk, the SNG2025 recommend abstinence [59,62].

This context underscores the public health relevance of the dietary gaps identified in Section 4.2 (legumes, whole grains, nuts, and seeds ↑ and red/processed meat, free sugars/SSBs, sodium, and alcohol ↓) and provides the rationale for the policy considerations discussed in Section 4.5.

### 4.5. Environmental Context

Given the contribution of the food system to GHG emissions and resource use, plant-forward shifts of the type specified by the SNG2025 are expected to reduce dietary footprints while supporting health [3,19,39]. Quantitative health and environmental impact modelling are beyond the scope of this paper and will be reported in the companion analysis (Article 5).

### 4.6. Interpretation and Implications

Dietary guidelines are a cornerstone of coherent food policy. They are increasingly embedded across food systems to support healthier, lower-impact consumption patterns, which are consistent with the FAO/WHO Guiding Principles for Sustainable Healthy Diets [63]. The evidence indicates that effective implementation requires coordinated multisector action and strong political commitment [64].

Healthy public food procurement and service standards can support healthier default options in schools, workplaces, and hospitals [65]. Affordability remains a binding constraint: global evidence highlights the high and unequal cost of healthy diets and the need for fiscal and social protection measures alongside supply-side action [66]. Retail and out-of-home environments have been shown to be influenced by strategic placement, pricing, and promotion “nudges”, particularly for families and adolescents [67]. Evidence collated in the NOURISHING framework shows that coordinated policy packages—reformulation, front-of-package labelling, marketing controls, retail interventions, and public procurement—are more effective than isolated measures [68]. In parallel, a workforce and public education system is needed to support nutritionally adequate plant-forward diets, especially in settings with traditionally high livestock consumption [63,65].

The SNG2025 translate a plant-forward paradigm into quantified targets for key health- and sustainability-relevant foods—whole grains, pulses (including soy foods), and nuts/seeds—within the EAT–Lancet alignment, filling gaps left by previous Slovenian FBDGs [3,7]. Given the observed population-level gaps (Table 2)—insufficient intake of fibre-rich plant foods and excessive consumption of red/processed meat, salt, free sugars/SSBs, UPFs, and alcohol—the SNG2025 provide a structured benchmark for implementation. These findings should, therefore, be interpreted as alignment with a newly defined policy benchmark rather than evidence of deterioration in dietary quality per se, given that underlying dietary patterns remain largely unchanged. Their implementation would require multisector collaboration and the embedding of preventive nutrition in national agendas, consistent with the recommendations of the Strategic Council for Nutrition [2]. With the low baseline adoption of plant-forward patterns in Slovenia (≈3.3% vegetarian and ≈0.4% vegan adults) [56], progress will depend on mainstream, population-wide measures (default plant-forward options, reformulation, and procurement standards) rather than relying on niche dietary adoption alone.

### 4.7. Policy-Relevant Considerations Based on Quantified Gaps

Priority considerations aligned with quantified gaps are listed below.

*Protein sources*. The quantified gaps indicate insufficient consumption of legumes (including soy foods), nuts, and seeds and excessive consumption of total and red/processed meat relative to the SNG2025 upper limits. These patterns point to the relevance of shifts towards plant-based protein sources, including the revalorisation of traditional plant-based dishes and measures that improve the affordability, accessibility, and acceptability of these options in public food environments and institutional catering.

*Plant-forward adequacy*. For adults who do not consume milk/dairy or fish, the observed alignment patterns highlight the importance of ensuring access to nutritionally adequate alternatives, including (i) unsweetened, calcium- and vitamin-B12-fortified dairy alternatives (including iodine and vitamin D fortification where available), supported by clear labelling and procurement standards, and (ii) a source of EPA + DHA ≈ 250 mg/day, preferably from oily fish or, if fish are not consumed, algal oil. Seasonal vitamin D supplementation and practical guidance on nutrient bioavailability (e.g., iron–polyphenol timing and consumption of vitamin C with plant iron) are consistent with national guidance and the assumptions underlying the alignment analysis [31,32].

*Fats and oils*. The quantified gaps indicate excessive intake of added fats and an unfavourable fat composition, particularly animal fats. Within the SNG2025 framework, alignment is shaped by both the total amount of added fats and their qualitative composition. From an interpretative perspective, the observed patterns suggest that shifts from animal fats and tropical oils towards nontropical plant oils and higher intakes of nuts and seeds would substantially affect alignment with targets. This shift is vital because animal fats are substantial sources of SFAs, and butter/meat are the primary sources of residual (ruminant) TFAs in Slovenia; reducing their use would, therefore, reduce both SFA and TFA exposure [20]. The magnitude of excess is greater in men, indicating the sex-specific relevance of these gaps.

*Salt and free sugars*. UPFs are recognised as major contributors to the population’s intake of hidden salt, free sugars, SFAs, and TFAs. Accordingly, both the SNG2025 and previous Slovenian FBDGs highlight the importance of minimising these foods in the context of sodium-reduction and reformulation strategies (see Supplementary Box S1 in [4] for practical label rules and swaps). The observed excesses are consistent with international evidence showing that progress towards population targets for salt (<5 g/day) and free sugars (<5% energy) is strongly influenced by product reformulation, food environments, and beverage consumption patterns [22,24]. This adjustment is a life-course issue: Slovenian adolescents already derive approximately 16–17% of their energy from free sugars [58]. In adults, the primary sources are beverages, cakes/muffins/pastry, and sugars/honey [69]. Slovenian school trials demonstrate the greatest SSB reductions when social marketing communication is combined with environmental change [70].

*Alcohol*. Excess alcohol intake contributes to misalignment with SNG2025 targets, which is consistent with evidence that population-level reductions in alcohol-related harm are driven mainly by structural policy measures and brief primary-care interventions [59].

*Monitoring considerations*. The quantified gaps highlight the need for sustained population-level monitoring, including 24-h urinary sodium surveys, improved capture of sweetening of tea/coffee in dietary assessments, and tracking of UPF exposure using sales-weighted nutrient data across retail and food service settings.

By quantifying targets and offering three plate models that accommodate diverse preferences (including WFPB), the SNG2025 provide a measurable, implementable framework that was absent from prior Slovenian FBDGs, enabling baseline-to-target monitoring, targeted gap reduction (e.g., legumes/nuts ↑ vs. meat/eggs ↓), and alignment with national noncommunicable disease and climate–biodiversity goals [3,8,71]. These monitoring priorities align with national recommendations for Slovenia, including routine surveillance, nutrition education, reformulation, and public procurement standards [2,8].

### 4.8. Implementation Pathways and Dissemination

#### 4.8.1. Culturally Anchored Implementation (Traditional Dishes)

Implementation pathways may build on Slovenia’s plant-forward culinary heritage, where traditional dishes based on whole grains and pulses, such as *ajdovi žganci* (buckwheat spoonbread), *ričet* (barley–bean stew), *jota* (sauerkraut/turnip and bean stew), *matevž* (bean–potato mash), *prosena kaša* (millet groats), and buckwheat crêpes, are well established and culturally familiar. These dishes provide familiar dietary patterns that align with international recommendations that emphasise whole grains, legumes, vegetables, and fruits as dietary staples.

From an implementation perspective, their inclusion in public food provisions, retail promotions, and culinary education illustrates how quantified targets for legumes and whole grains in the SNG2025 can be operationalised within existing food cultures. The evidence suggests that default plant-forward options in institutional settings, combined with recipe optimisation (e.g., lower salt and animal fat contents) and procurement aligned towards whole grains, pulses, and nontropical plant oils, may facilitate adherence to plant-forward dietary frameworks without requiring radical dietary change [72,73].

#### 4.8.2. Dissemination and Behaviour-Change Levers

Public uptake of the SNG2025 is likely to depend on sustained, targeted dissemination across mass/social media, supported by digital tools, cookbooks, and coordinated campaigns [6,74]. Evidence from behavioural nutrition research indicates that small, low-effort choice-architecture interventions “nudges” produce modest but consistent effects at the population level [75,76], particularly when combined with improvements in food literacy (planning, food selection, and preparation skills) [77,78].

Recent studies further suggest that personalised digital decision support tools can improve engagement and adherence, especially when guidance is tailored to individual dietary patterns and constraints [79,80,81].

Across intervention trials, the most effective approaches combine feasible substitutions (e.g., legume-forward meals and plant-based alternatives) with post-selection reinforcement, increasing the likelihood that behavioural changes translate into measurable intake differences in intake [82,83,84,85]. In this context, dissemination strategies that explicitly reference the quantified dietary gaps identified in this study (Figure 1) may help prioritise the most impactful dietary shifts, while future evaluations should report effect sizes, sustainability indicators, and longer-term adherence to support real-world scalability.

#### 4.8.3. Capacity-Building and Education

Alignment with the SNG2025 will partly depend on capacity-building across health, food, and education systems. Evidence from the implementation of previous guidelines indicates that updating curricula and accredited training, including continuing medical education and professional development, supports the consistent application of plant-forward dietary guidance. Practical tools such as simple counting rules (MCE and swaps) and the use of culturally familiar Slovenian traditional dishes may facilitate the translation of quantified targets into routine practice and communication [2,3,8].

### 4.9. Life-Course Perspective

Although this analysis targeted adults (18–64 years), nationally representative studies in Slovenian adolescents reveal similar directional gaps (low intake of fibre and fruits/vegetables and high intake of meat and meat products, UPFs, free sugars/SSBs SFA, and sodium), indicating shared system-level drivers such as food availability, product formulation, and marketing/placement [57,58]. Thus, the adult-focused gaps identified here are likely to have relevance across the life course. The development of child/adolescent SNGs, harmonised with the SNG2025 principles, remains an important next step.

### 4.10. Strengths

*National representativeness + biomarker anchor*. Si. Menu 2017/18 (for adults) paired with 24-h urinary sodium levels (2022/23) strengthens internal validity for salt intake and reduces shared-method bias.

*Sex-specific estimates*. Systematic male/female stratification reveals materially different gaps (meat, fats/oils, and water), enabling targeted actions.

*Target framework, scoring discipline, and visualisation*. We apply an explicit target taxonomy (≤ limits, ≥ targets, and range-type bands) to sharpen the classification and reveal policy levers; midpoint references (e.g., 250 mL of milk and 32 g of fish) are for context only (not used for adherence scoring), and the results are shown with EAT–Lancet-aligned 100% boundary figures for rapid interpretation.

*Optional categories coded as range-type*. Milk at 250 mL (0–500 mL/day) and fish at 32 g (0–64 g/day) are treated as a range type; any intake within the band, including zero, is fully compliant with the SNG2025, reflecting the intended flexibility (e.g., plant-forward dietary patterns).

*Key drivers of the dietary gap and priority intervention targets*. A small set of groups (legumes, nuts/seeds, vegetables, meat, salt, and free sugars) explains most of the “dietary gap,” guiding efficient interventions.

*Actionability by design*. The results translate into procurement/catering standards, portion upper limits, and menu engineering, bridging evidence to implementation.

*Transparency about data gaps*. Missing domains (whole-grain quality, soy category, cooking methods, and % E from SFAs) are explicitly stated, improving credibility and guiding data collection.

*Consistency with international guidance*. Directions of change (↓ red/processed meat, salt, free sugars and ↑ vegetables/legumes/nuts) align with the WHO/EU/EAT–Lancet recommendations, supporting external validity.

*Reproducible methodology*. Mapping rules (food group → target type → % limit/target) are explicit and reusable for future waves and monitoring.

### 4.11. Limitations

At the analytical level, the absence of inferential statistical testing represents an inherent limitation of the analytical design and reflects the use of aggregated surveillance data rather than individual-level dietary records. Because the analysis was based on population-level means without measures of variability, formal hypothesis testing and uncertainty estimation were not feasible. As a result, the findings should be interpreted as descriptive and as normative alignment with predefined guideline targets rather than as statistical inference regarding individual-level dietary behaviour or causal associations. Nevertheless, this approach is well suited to the study objective of quantitatively benchmarking population dietary intake against national and international dietary frameworks. Accordingly, the results describe population-level alignment with dietary benchmarks rather than within-population variability or individual adherence.

*Survey period*. Key constraints include the survey period (2017/18); changes occurring after this period may not be captured.

*Measurement granularity*. Several domains lack detail (e.g., whole-grain quality, soy foods as a distinct group, herbs/spices, and % energy from SFAs), limiting direct alignment with the SNG2025 benchmarks for those rows in Table 1 and Table 2.

*Self-reporting bias*. Multiple components rely on self-reported intake, reducing precision and cross-row comparability, as shown in Table 1 and Table 2.

*Cooking methods were not recorded*. Although the SNG2025 specify boiled/baked (no deep-frying) potatoes and the avoidance of animal fats, the Si. Menu does not code cooking methods; some reported “potatoes” or “dishes with fats/oils” may include fried forms. This lack of reporting underscores the need for cooking-method standards in catering/procurement (no deep-frying; no butter/lard/ghee; and minimal plant oils) plus portion guidance.

Taken together, these limitations of the best available food intake data in Slovenia (Si. Menu 2017/18) affect precisely those food groups central to the SNG2025 framework, including whole grains, fats, and dairy. As a result, some degree of systematic misclassification at the food group level cannot be excluded and may influence both the direction and the magnitude of estimated shortfalls and excesses. Consequently, the results should be interpreted with caution, particularly regarding the exact magnitude of deviations from SNG2025 targets, while the overall direction of misalignment remains robust.

*Population scope*. Adults aged 65–74 years were excluded to maintain focus and length; the findings may not be generalisable to older adults.

Methodological considerations affecting the interpretation. Our methodological choices influence the magnitude and visual emphasis of the observed excesses and shortfalls and should, therefore, be interpreted with caution. First, all targets and intakes were harmonised to a reference energy intake of 2500 kcal/day, validated at the population level, but did not reflect individual energy and nutrient requirements, which vary by age, sex, body size, physical activity, and health status. Second, weekly upper limits (e.g., for total meat and eggs) are reported as daily equivalents to enable direct comparisons between observed intakes and guideline thresholds. While this approach facilitates comparison and visualisation, it may amplify apparent deviations when the results are presented as percentages and should not be interpreted as prescriptive daily limits. Third, aggregating added fats and fat-rich foods into a single category is appropriate at the level of total fat intake but may obscure food-specific dietary patterns. Fourth, the inclusion of eggs “including from foods” reflects scientific precision while increasing complexity for individual-level interpretation. Alternative operationalisations, such as presenting weekly limits exclusively on a weekly basis or further disaggregating fat sources, would yield different visual representations of alignment without altering the overall conclusions. Taken together, these considerations indicate that the reported magnitudes primarily serve comparative and policy-monitoring purposes rather than individual dietary assessment and provide a qualitative sensitivity perspective on the robustness of the observed misalignment.

### 4.12. Future Research

*Comparative analyses*. (i) Quantify the health and environmental consequences of shifting from current intakes to the SNG2025 using a comparative risk assessment (clinical risk factors and events) and life-cycle footprints (GHG, land, and water); detailed methods for different scenarios and daily plate inputs are reported in the companion paper. (ii) Benchmark the SNG2025 quantitatively against the EAT–Lancet reference ranges [3] and against early adopters’ FBDGs that integrate health and sustainability [47,48,49,50,51,86,87]. (iii) Compare the lifestyle characteristics of the SNG2025 with previous Slovenian FBDGs.

*Alignment metrics and methods (builds on Table 1 and Table 2)*. (iv) Improve food-group granularity in national surveys to resolve “no data” domains (whole-grain quality, soy foods, herbs/spices, and % E from SFAs) and validate UPF exposure with sales-weighted nutrient data across retail and food services [8]. (v) Enhance biomarker panels for surveillance (repeat 24-h urinary sodium measurements and consider fatty acid profiles for SFAs/TFAs) and standardise adherence classification rules (daily vs. weekly upper limits and energy scaling). (vi) Conduct age- and equity-stratified alignment: extend analyses to older adults (65–74 y), children and adolescents, and examine socioeconomic, regional, and urban–rural gradients relevant for targeted implementation [88,89].

*Policy evaluation*. (vii) In real-world settings, prioritise clustered policy packages directly mapped to the quantified gaps (protein intake shifting towards legumes/nuts; standardised WFPB options; sodium and free sugar reformulation/procurement; and SSB/marketing measures), with implementation-science endpoints (fidelity, reach, acceptability, and equity). Detailed policy menus are discussed elsewhere and are not repeated here [2].

## 5. Conclusions

This study provides the first quantitative alignment of nationally representative Slovenian dietary intake data with the SNG2025 using an integrated health-, sustainability- and equity-oriented reference framework aligned with the EAT–Lancet planetary health diet. The results demonstrate a systematic misalignment between observed adult dietary patterns and the SNG2025 targets, characterised by insufficient intake of key protective plant-based food groups (vegetables, pulses/legumes, whole grains, and nuts/seeds) alongside the excessive consumption of total and red/processed meat, sodium, free sugars (particularly from SSBs), and alcohol.

A major scientific contribution of this work lies in its methodological approach. By operationalising qualitative dietary guidelines into explicit quantitative targets (upper limits, minimum targets, and permissible intake ranges) and applying them consistently to population-level intake data, this analysis moves beyond a descriptive reporting of guidelines. The target-based framework enables the transparent classification of alignment, identification of priority food-group gaps, and sex-specific interpretation while explicitly accommodating plant-forward dietary patterns, including vegetarian and WFPB diets.

The use of nationally representative dietary data, supported by biomarker-based sodium measurements, strengthens confidence in the identified dietary gaps. While uncertainty remains regarding precise absolute intakes in selected domains because of known data limitations, the overall pattern of misalignment is robust and consistent with findings from other European countries that have recently adopted health- and sustainability-integrated dietary guidelines.

The analytical framework presented in our study is replicable internationally. Countries with existing dietary intake surveys can apply the same harmonisation rules, target taxonomy, and alignment metrics to assess consistency between observed diets and newly adopted healthy, sustainable, and equitable dietary guidelines. This approach enables cross-country comparability, longitudinal monitoring, and evaluations of guideline implementation without reliance on modelling assumptions.

In conclusion, this study demonstrates that integrating health, environmental sustainability, and equity into a single, quantitative dietary assessment framework provides a clearer and more policy-neutral scientific basis for evaluating population diets. This approach offers a transferable tool for monitoring alignment with sustainable dietary guidelines and for advancing comparative nutrition research at the national and international levels.

## Data Availability

No new data were created or analysed in this study. Data sharing does not apply to this article.

## Supplementary Materials

The following supporting information can be downloaded at https://www.mdpi.com/article/10.3390/foods15030524/s1, Supplementary Table S1. Core Working Group (Writing Group) for the SNG2025.

## Abbreviations

DHA	docosahexaenoic acid
EPA	eicosapentaenoic acid
EAT–Lancet	EAT–Lancet Commission planetary health diet (reference)
FBDGs	food-based dietary guidelines
MCE	milk-calcium equivalent
SFAs	saturated fatty acids
SSBs	sugar-sweetened beverages
TFAs	trans fatty acids
UPFs	ultra-processed foods
SNG2025	Slovenian Nutrition Guidelines 2025
WFPB	whole-food, plant-based
WHO	World Health Organization
