# Expression of Concern: Fidler Mis et al. The Slovenian Nutrition Guidelines 2025: A Comparison with the Prior Slovenian FBDG, Dietary Intake, and the EAT–Lancet Diet. *Foods* 2026, *15*, 524

**DOI:** 10.3390/foods15112009

**Published:** 2026-06-04

**Authors:** Foods Editorial Office

**Affiliations:** MDPI, St. Alban-Anlage 66, 4052 Basel, Switzerland; foods@mdpi.com

With this notice, the *Foods* Editorial Office alerts the readers of multiple concerns related to this article [1] cited below. Following publication, concerns were raised to the Editorial Office, and the authors of this publication have been contacted for further clarification. The investigation is being coordinated by the Editorial Office with the involvement of the Editor-in-Chief.

Once this process is complete, the Editorial Office will provide an update on this situation and announce any post-publication modification deemed to be necessary, as per MDPI’s Updating Published Papers policy.
