# Supplementary material for: The Slovenian Nutrition Guidelines 2025: A Comparison with the Prior Slovenian FBDG, Dietary Intake, and the EAT–Lancet Diet
Source: Foods. 2026 Feb 3;15(3):524. doi: 10.3390/foods15030524 (PMC12897075; doi:10.3390/foods15030524)
Supplement: Supplementary file 1 [file foods-15-00524-s001.zip › foods-4103991-supplementary.pdf]

Supplementary Table S1. Core Working Group (Writing Group) for the SNG2025.

Members with affiliation superscripts:

Nataša Fidler Mis, BSc, MSc, PhD[1*]

Martina Bavec, MSc, PhD[2]

Boštjan Jakše, BSc, PhD[3]

Borut Jug, MD, PhD[4,5]

Samo Kreft, MPharm, PhD[6]

Žiga Malek, BSc, PhD[7,8, ***]

Nina Mikec, MSc, PhD[9]

Nana Turk, BSc, MSc[10]

Ana Vovk, Prof., PhD[11]

Zlatko Fras, MD, PhD, FRCP (Lond), FESC, FACC[4,5,12,**]

Affiliations:

[1] Ministry of Health, Ljubljana, Slovenia; Independent Researcher, Ljubljana, Slovenia

[2] Faculty of Agriculture and Life Sciences, University of Maribor, Maribor, Slovenia

[3] Independent Researcher, Kranjska Gora, Slovenia

[4] Department of Vascular Disease, University Medical Centre Ljubljana, Ljubljana, Slovenia

[5] Faculty of Medicine, University of Ljubljana, Ljubljana, Slovenia

[6] Faculty of Pharmacy, University of Ljubljana, Ljubljana, Slovenia, Independent Researcher, Divača, Slovenia

[7] International Institute for Applied Systems Analysis (IIASA), Laxenburg, Austria

[8] Biotechnical Faculty, University of Ljubljana, Ljubljana, Slovenia

[9] Department of Molecular and Biomedical Sciences, Jožef Stefan Institute, Ljubljana, Slovenia; Independent Researcher, Ljubljana

[10] Central Medical Library, Faculty of Medicine, University of Ljubljana, Ljubljana, Slovenia

[11] Faculty of Arts, University of Maribor, Maribor, Slovenia

[12] Division of Medicine, Centre for Preventive Cardiology, University Medical Centre Ljubljana, Ljubljana, Slovenia

Footnotes:

[*] Lead author during the first half of the guideline development

[**] Lead author during the second half of the guideline development

[***] Lead author of Part II (sustainability) of the guideline development
